# Bone marrow mesenchymal stem cell-derived extracellular vesicles containing miR-181d protect rats against renal fibrosis by inhibiting KLF6 and the NF-κB signaling pathway

**DOI:** 10.1038/s41419-022-04875-w

**Published:** 2022-06-07

**Authors:** Shi-Jun Wang, Zhen-Zhen Qiu, Fu-Wei Chen, An-Li Mao, Jun-Chao Bai, Ye-Jing Hong, Zhong-Pan Zhang, Wu-An Zhu, Zhi-Wei Zhang, Hao Zhou

**Affiliations:** 1 Surgery Department of Traditional Chinese Medicine, The Affiliated People’s Hospital of Fujian University of Traditional Chinese Medicine (The People’s Hospital of Fujian Province), 350004 Fuzhou, PR China; 2 Department of Physical Education, Minjiang University, 350108 Fuzhou, PR China; 3 Department of Urology, The Fourth Affiliated Hospital, International Institutes of Medicine, Zhejiang University School of Medicine, 322000 Yiwu, PR China; 4 Department of Nephrology, The Fourth Affiliated Hospital, International Institutes of Medicine, Zhejiang University School of Medicine, 322000 Yiwu, PR China; 5 Department of Research, Beijing Zhong Jian Dong Ke Company, 100176 Beijing, PR China

**Keywords:** Cell biology, Biotechnology

## Abstract

Recent studies have investigated the ability of extracellular vesicles (EVs) in regulating neighboring cells by transferring signaling molecules, such as microRNAs (miRs) in renal fibrosis. EVs released by bone marrow mesenchymal stem cells (BMSCs) contain miR-181d, which may represent a potential therapy for renal fibrosis. miR-181d has been speculated to regulate Krüppel-like factor 6 (KLF6), which activates the nuclear factor-kappa B (NF-κB) signaling pathway. Luciferase assays were performed to confirm the relationship between miR-181d and KLF6. Gain- and loss-of-function studies in vivo and in vitro were performed to assess the effect of BMSC-derived EVs (BMSC-EVs), which contained miR-181d, on KLF6, NF-κB, and renal fibrosis. Transforming growth factor-β (TGF-β)-induced renal tubular epithelial HK-2 cells were treated with EVs derived from BMSCs followed by evaluation of collagen type IV α1 (Col4α1), Collagen I and α-smooth muscle actin (α-SMA) as indicators of the extent of renal fibrosis. Renal fibrosis was induced in rats by unilateral ureteral obstruction (UUO) followed by the subsequent analysis of fibrotic markers. BMSC-EVs had higher miR-181d expression. Overexpression of miR-181d correlated with a decrease in KLF6 expression as well as the levels of IκBα phosphorylation, α-SMA, Col4α1, TGF-βR1 and collagen I in HK-2 cells. In vivo, treatment with miR-181d-containing BMSC-derived EVs was able to restrict the progression of fibrosis in UUO-induced rats. Together, BMSC-EVs suppress fibrosis in vitro and in vivo by delivering miR-181d to neighboring cells, where it targets KLF6 and inhibits the NF-κB signaling pathway.

## Introduction

Chronic kidney disease is frequently characterized by renal cell fibrosis resulting in the progressive loss of kidney function [1]. Renal fibrosis is featured by a large accumulation of extracellular matrix within the interstitium [2]. Unfortunately, existing treatments only slow the progression of renal fibrosis but do not cure the disease [3]. The identification of morphological markers as well as early interventions is important for improving graft function and survival [4].

Interestingly, extracellular vesicles (EVs), which include exosomes and microvesicles, represent a potential drug delivery system in treatment of renal fibrosis [5]. EVs contain lipids and multiple functional transcripts such as messenger RNAs (mRNAs), microRNAs (miRs), long non-coding RNAs, and genomic DNA that are produced by one cell and delivered to neighboring target cells. The transfer of signaling molecules *via* EVs induces transient or persistent phenotypic alterations in recipient cells [6].

Mesenchymal stem cells (MSCs) are adult stromal cells capable of differentiating into a variety of cell types. They also modulate normal cellular functions and the development of disease by transferring regulatory factors *via* EVs [7]. It is noteworthy that allogeneic transplantation of rat bone marrow mesenchymal stromal cells (BMSCs) can inhibit renal fibrosis by means of microvascular protection [8]. Based on our bioinformatics analysis, we determined that miR-181d expression was highly expressed in BMSC-derived EVs (BMSC-EVs) from renal healthy tissues, but was downregulated in EVs from fibrotic renal tissues. A previous study has shown that EVs containing miR-181d are an important regulator of renal blood flow [9].

Krüppel-like factor 6 (KLF6) was speculated as a target of miR-181d, and is a DNA-binding protein that contains three zinc-fingered motifs important in the regulation of renal fibrosis pathogenesis [10]. As previously reported, KLF6 is upregulated in fibrotic kidneys [11]. Our bioinformatics analysis indicated that KLF6 co-activates nuclear factor-kappa B (NF-κB) by regulating p65-dependent transcription of downstream genes [12]. Notably, the downregulation of NF-κB pathway enhances telbivudine-mediated attenuation in renal fibrosis and inflammatory reactions after unilateral ureteral obstruction (UUO) [13] and Xiexin decoction-induced suppression of renal fibrosis in diabetic mice [14]. Based on this information, we tested whether miR-181d-containing EVs derived from BMSCs could suppress renal fibrosis by inhibiting KLF6 and the NF-κB signaling pathway.

## Results

### BMSC-EVs were isolated and transferred to human renal tubular epithelial cells

Mesenchymal stem cell markers CD29, CD44, and CD73 were highly expressed, while hematopoietic markers such as CD34, CD45, and human leukocyte antigen D-related (HLA-DR) were poorly expressed (Supplementary Fig. 1A), indicating the successful isolation of BMSCs.

EVs isolated from human BMSCs (hBMSCs) were measured by dynamic light scattering (Supplementary Fig. 1B) and transmission electron microscopy (Supplementary Fig. 1C), respectively. The distribution of EV diameter was between 30 and 100 nm and the EVs were cup-shaped or spherical. Furthermore, the results of Western blot analysis (Supplementary Fig. 1D) revealed that EV maker proteins CD63 and TSG101 were highly enriched in the isolated EVs, when compared to cell lysates. Uptake of EVs resulted in HK-2 cells being labeled with PKH67 and appearing green when observed using a fluorescence microscope (Supplementary Fig. 1E). Together, BMSC-EVs could be successfully isolated and effectively shuttled to renal tubular epithelial cells.

### BMSC-EVs inhibited renal fibrosis both in vitro and in vivo

**Fig. 1:**
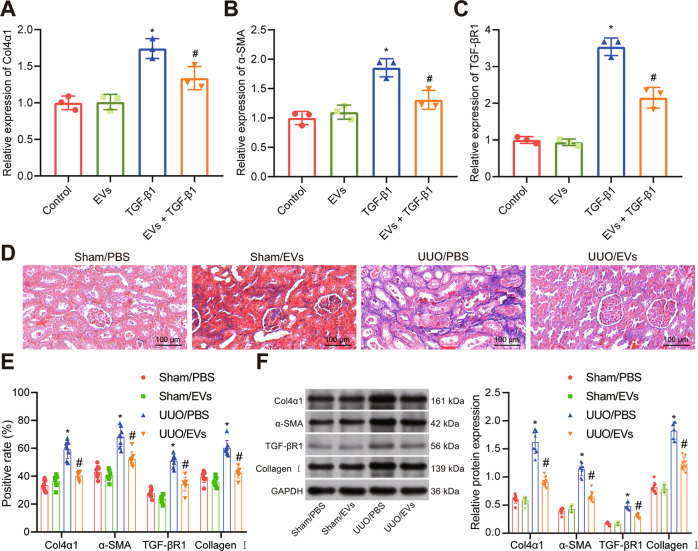
BMSC-EVs inhibit renal fibrosis in vitro and in vivo. **A** RT-qPCR measurement of Col4α1 expression in HK-2 cells after treatment with TGFβ-1 and BMSC-EVs. **B** RT-qPCR measurement of α-SMA expression in HK-2 cells after treatment with TGFβ-1 and BMSC-EVs. **C** RT-qPCR measurement of TGF-βR1 expression in HK-2 cells after treatment with TGFβ-1 and BMSC-EVs. In the panels of (**A**, **B**, **C**) *p < 0.05 *vs*. control. ^#^*p* < 0.05 *vs*. TGFβ-1. **D** Masson’s trichrome staining for the deposition of collagen in the renal tissues from UUO rats treated with BMSC-EVs (*n* = 8). **E** Immunohistochemistry assay of Col4α1, α-SMA, TGF-βR1 and collagen I in renal tissues from UUO rats treated with BMSC-EVs (*n* = 8). **F** Western blot analysis of Col4α1, α-SMA, TGF-βR1, and collagen I in renal tissues from UUO rats treated with BMSC-EVs. In the panels of (**E**, **F**) *p < 0.05 *vs*. rats receiving sham operation and injected with PBS. ^#^*p* < 0.05 *vs*. rats undergoing UUO surgery and injected with PBS. The cell experiment was repeated three independent times.

In order to determine the function of EVs on renal fibrosis, we stimulated HK-2 cells with transforming growth factor-β1 (TGFβ-1) for 72 h, and then cultured them with BMSC-EVs. Co-culture with BMSC-EVs diminished the expression of Col4α1 (Fig. 1A), α-smooth muscle actin (α-SMA) (Fig. 1B) and TGF-βR1 (Fig. 1C) in HK-2 cells compared to their expression in control HK-2 cells.

BMSC-EVs were injected *via* tail vein into UUO rats and renal sections were prepared and stained with Masson’s trichrome staining. The rats injected with BMSC-EVs had a notable decline in collagen deposition in renal tissues compared with the UUO renal tissues from rats that did not receive BMSC-EVs (sham-operated rats) (Fig. 1D). Immunohistochemistry analysis showed that the expression of Col4α1, α-SMA, and collagen I was markedly lower in the renal tissues of UUO rats treated with EVs than those treated with phosphate buffer saline (PBS) and Western blot analysis of Col4α1, α-SMA, TGF-βR1, and collagen I protein expression confirmed this (Fig. 1E, F). Collectively, BMSC-EVs are capable of inhibiting rat renal fibrosis both in vitro and in vivo.

### Transfer of miR-181d by BMSC-EVs inhibited fibrosis of HK-2 cells

**Fig. 2:**
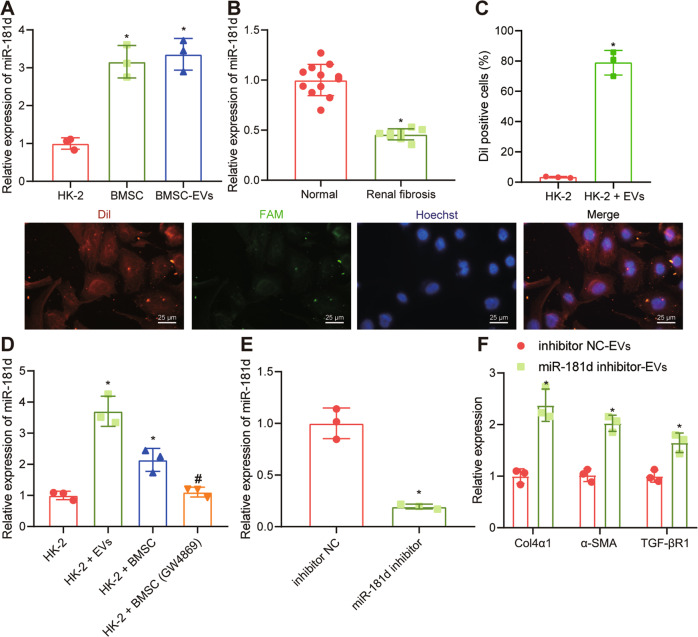
BMSC-EVs transfer miR-181d to inhibit fibrosis in HK-2 cells. **A** miR-181d expression in HK-2 cells, BMSCs or BMSC-EVs. *p < 0.05 *vs*. HK-2 cells. **B** RT-qPCR analysis of miR-181d expression in renal tissues from patients with renal fibrosis (*n* = 8; two cases at stage I, three cases at stage II, two cases at stage III, and one case at stage IV) or healthy controls (*n* = 12). *p < 0.05 *vs*. healthy controls. **C** Immunofluorescence detection of miR-181d transfer by BMSC-EVs into HK-2 cells. *p < 0.05 *vs*. HK-2 cells. **D** RT-qPCR analysis of the expression of miR-181d in HK-2 cells after co-culture of EVs and BMSCs or those treated with GW4869. *p < 0.05 *vs*. HK-2 cells. ^#^*p* < 0.05 *vs*. HK-2 cells co-cultured with BMSCs. **E** RT-qPCR analysis of miR-181d expression in BMSCs in the presence of miR-181d inhibitor. *p < 0.05 *vs*. inhibitor NC. **F** RT-qPCR analysis of Col4α1, α-SMA, and TGF-βR1 expression in HK-2 cells in the presence of miR-181d inhibitor-EVs. *p < 0.05 *vs*. inhibitor NC-EVs. The cell experiment was repeated three independent times.

Next, we set out to test the hypothesis that BMSC-EVs might inhibit the fibrosis of renal tubular epithelial cells by transferring a specific miRNA. Accordingly, we found that miR-181d expression was enriched in BMSC-EVs from the kidney using the EVmiRNA database (http://bioinfo.life.hust.edu.cn/EVmiRNA#!/) and this finding was verified by RT-qPCR (Fig. 2A). At the same time, we found in the renal tissues of patients with renal fibrosis that the expression of miR-181d was reduced (Fig. 2B). In order to study whether miR-181d was transferred to HK-2 cells by BMSC-EVs, we first transfected BMSCs with FAM-miR-181d (green), and then labeled the secreted EVs with DiL (red). The EVs were subsequently co-cultured with TGFβ-1-stimulated HK-2 cells labeled with Hoechst (blue) for 6 h. The red and green signals were witnessed in HK-2 cells co-cultured with EVs (Fig. 2C). The expression of miR-181d was notably increased in HK-2 cells co-cultured with EVs and BMSCs compared with that in the HK-2 cells cultured with only BMSCs. In contrast, treatment with GW4869 (inhibitor of EV secretion) reversed this effect (Fig. 2D). These results confirm that the BMSC-EVs can transfer miR-181d into HK-2 cells.

Moreover, it was displayed that miR-181d inhibitor diminished the expression of miR-181d in the BMSCs (Fig. 2E). In addition, RT-qPCR results showed an upregulation of Col4α1, α-SMA and TGF-βR1 expression in HK-2 cells after treatment with EVs containing miR-181d inhibitor (Fig. 2F). This suggests that BMSC-EVs can inhibit renal fibrosis by delivering miR-181d.

### Overexpression of miR-181d inhibited renal fibrosis in vivo and in vitro

**Fig. 3:**
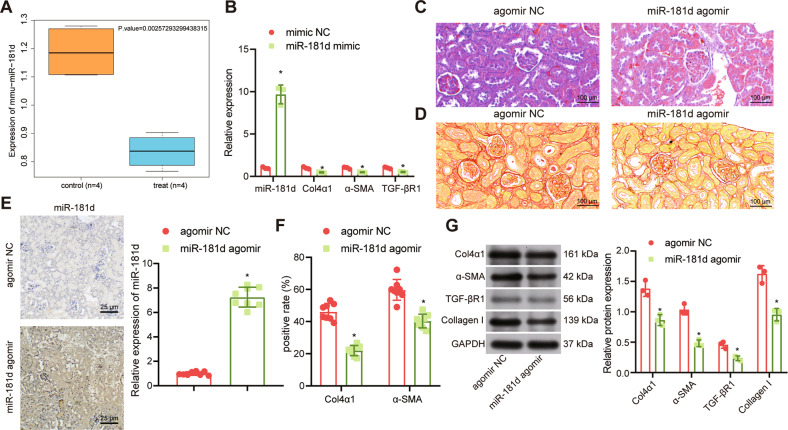
Overexpression of miR-181d inhibits renal fibrosis in vivo and in vitro. **A** A box plot of the expression of miR-181d in GSE125305 dataset. The blue box on the left indicates the expression in normal control samples (*n* = 9), and the red box on the right indicates the expression in renal fibrosis samples (*n* = 9). **B** RT-qPCR analysis of α-SMA, Col4α1, and TGF-βR1 expression in HK-2 cells. **C** Masson’s trichrome staining of collagen deposition in UUO rats after treatment with BMSC-EVs (*n* = 8). **D** Sirius red staining of collagen deposition in renal tissues from UUO rats after treatment with miR-181d agomir (*n* = 8). **E** In situ hybridization to assess miR-181d expression in renal tissues from UUO rats after injection of miR-181d agomir (*n* = 8). **F** Immunohistochemistry assay of Col4α1 and α-SMA in renal tissues from UUO rats after injection of miR-181d agomir (*n* = 8). **G** Western blot analysis of Col4α1, α-SMA/TGF-βR1 and collagen I expression in renal tissues from UUO rats after injection of miR-181d agomir. *p < 0.05 *vs*. rats treated with agomir NC. The cell experiment was repeated three independent times.

The GSE125305 dataset was obtained from the Gene Expression Omnibus (GEO) database (https://www.ncbi.nlm.nih.gov/gds) and analyzed for miR-181d expression. miR-181d expression was diminished in EVs during renal injury (Fig. 3A). Renal injury samples in GSE125305 dataset were characterized by renal fibrosis. RT-qPCR analysis of mRNA from HK-2 cells stimulated with TGFβ-1 and transfected with synthetic miR-181d analog showed an increase in miR-181d expression and a marked decrease in α-SMA, Col4α1, and TGF-βR1 expression (Fig. 3B).

Next, Masson’s trichrome staining revealed that rats injected with miR-181d agomir had a notable decline in collagen deposition in renal tissues relative to the rats without injection (Fig. 3C). Sirius Red staining also showed similar results (Fig. 3D). In situ hybridization revealed a significant increase in miR-181d expression in the renal tissues of UUO rats after treatment with miR-181d agomir (Fig. 3E) while immunohistochemical assay indicated a reduction in Col4α1 and α-SMA expression (Fig. 3F). Western blot analysis also demonstrated lower Col4α1, α-SMA, TGF-βR1 and collagen I protein expression in the renal tissues of UUO rats injected with miR-181d agomir compared to those injected with agomir negative control (NC) (Fig. 3G). Collectively, overexpression of miR-181d can inhibit renal fibrosis in vitro and in vivo.

### miR-181d targeted KLF6

**Fig. 4:**
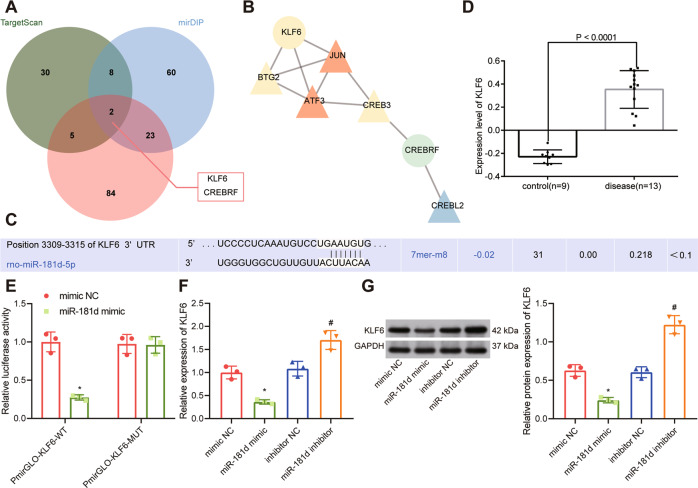
miR-181d targets KLF6. **A** A Venn diagram of the downstream targets of miR-181d predicted by the TargetScan, mirDIP and DIANA tool websites. **B** PPI networks for CREBRF and KLF6 and their related genes constructed using the String website. The circle indicates the input gene, and the triangle indicates the predicted related gene. The gene with redder color presented higher degree of correlation, while the gene with bluer color had lower degree of correlation. **C** The binding site between miR-181d and KLF6 was predicted using the Targetscan website. **D** A box line diagram based on the KLF6 expression data from the GSE36496 dataset. The blue box on the left represents the expression of normal control samples, and the red box on the right represents the expression of renal fibrosis samples. **E** miR-181d targeting of KLF6 is verified by dual luciferase reporter gene assay. *p < 0.05 *vs*. mimic NC. **F** RT-qPCR analysis of KLF6 mRNA expression in HK-2 cells after overexpression or silencing of miR-181d. *p < 0.05 *vs*. mimic NC. ^#^*p* < 0.05 *vs*. inhibitor NC. **G** Western blot analysis of KLF6 expression in HK-2 cells after overexpression or silencing of miR-181d. *p < 0.05 *vs*. mimic NC. ^#^*p* < 0.05 *vs*. inhibitor NC. The cell experiment was repeated three independent times.

In order to dissect out the downstream regulatory mechanism of miR-181d, we used the bioinformatics databases Targetscan (Cumulative weighted context++ score < −0.5; http://www.targetscan.org/vert_71/), mirDIP (Integrated Score > 0.8; http://ophid.utoronto.ca/mirDIP/) and Diana tools (miTG score > 0.95; http://diana.imis.athena-innovation.gr/DianaTools) to predict the downstream target genes of miR-181d and obtained 45, 93, and 114 downstream genes, respectively. A Venn diagram was created to identify genes that overlapped among the three tools. We found that only CREBRF and KLF6 were identified by all the analysis tools (Fig. 4A). Through the String website (https://string-db.org), we predicted the related genes of these two genes and constructed a protein-protein interactions (PPI) network. In addition, the core degree of KLF6 was found to be higher than that of CREBRF (Fig. 4B) based on the calculation from the analytical map drawn using the Cytoscape website (https://cytoscape.org). The binding site of miR-181d targeting KLF6 (Fig. 4C) was predicted using Targetscan. In addition, KLF6 was found to be overexpressed in renal fibrosis in the GSE36496 dataset, a GEO database profile (Fig. 4D).

Next, we verified the targeting relationship between miR-181d and KLF6 using the dual luciferase reporter gene assay. The co-transfection of miR-181d and PmirGLO-KLF6-wild type (WT) resulted in a notable decrease in fluorescence intensity compared with the co-transfection of miR-181d and PmirGLO-KLF6-mutant type (MUT) (Fig. 4E). Furthermore, KLF6 expression was diminished by miR-181d mimic, but it was notably increased after treatment with miR-181d inhibitor (Fig. 4F, G). The above results demonstrate that KLF6 is a downstream target of miR-181d, which can negatively regulate the expression of KLF6 in HK-2 cells.

### KLF6 promoted fibrosis of HK-2 cells through activation of the NF-κB signaling pathway

**Fig. 5:**
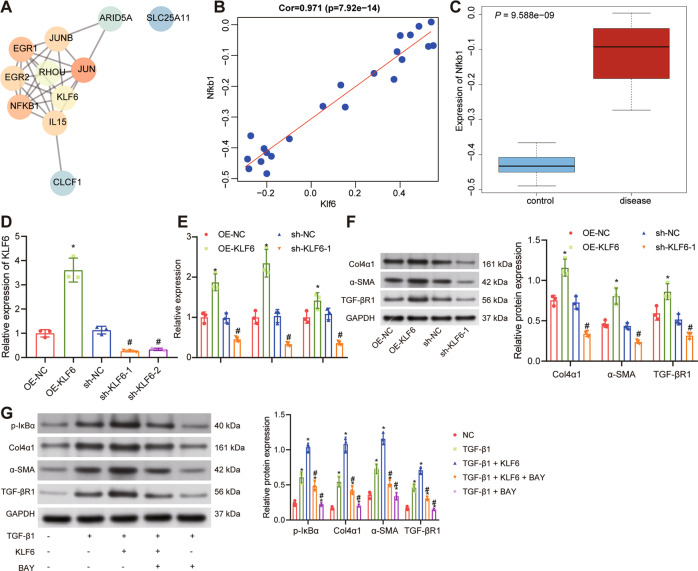
KLF6 promotes fibrosis of HK-2 cells by activating the NF-κB signaling pathway. **A** PPI network of KLF6 and its top ten related genes constructed using the String website. The redder the color of the gene, the higher the degree of correlation; the bluer the color of the gene, the lower the degree of correlation. **B** A diagram of the correlation between KLF6 and NFKB1 expression. **C** A box line diagram of NFKB1 expression data from the GSE36496 dataset. The blue box on the left represents the expression in normal control samples and the red box on the right represents the expression in renal fibrosis samples. **D** The transduction efficiency of oe-KLF6 and sh-KLF6 as verified by RT-qPCR in HK-2 cells. *p < 0.05 *vs*. oe-NC. ^#^*p* < 0.05 *vs*. sh-NC. **E** The effect of KLF6 on the mRNA expression of α-SMA, Col4α1, and TGF-β in HK-2 cells as detected by RT-qPCR. *p < 0.05 *vs*. oe-NC. ^#^*p* < 0.05 *vs*. sh-NC. **F** Western blot analysis of α-SMA, Col4α1 and TGF-β protein expression in HK-2 cells with KLF6 overexpression or knockdown. *p < 0.05 *vs*. oe-NC. ^#^*p* < 0.05 *vs*. sh-NC. **G** Western blot analysis of IκBα phosphorylation and α-SMA, Col4α1, and TGF-β protein expression in HK-2 cells after treatment with KLF6 overexpressing vector and BAY-11-7082. *p < 0.05 *vs*. NC. ^#^*p* < 0.05 *vs*. TGF-β-1 + KLF6. The cell experiment was repeated three independent times.

The correlation between KLF6 expression and other genes was calculated using the GSE36496 dataset (Supplementary Table 1). KLF6 and the top 10 related genes were selected, a PPI network map of these 11 genes was constructed using String, and the core degree was calculated using Cytoscape (Fig. 5A; Supplementary Table 1). Correlation diagram of KLF6 and NFKB1 expression in the GSE36496 dataset presented a positive correlation between KLF6 and NFKB1 expression (Fig. 5B). Meanwhile, NFKB1 was highly expressed in renal fibrosis samples (Fig. 5C) from the GSE36496 dataset.

Next, we constructed overexpression/silencing KLF6 plasmids and separately transfected them into HK-2 cells. RT-qPCR detection results confirmed the transfection efficiency and that short hairpin RNA (sh)-KLF6-1 exhibited the superior efficiency (Fig. 5D) and was thus selected for the subsequent experiments. In HK-2 cells induced with TGF-βR1, overexpression of KLF6 promoted the expression of α-SMA, Col4α1 and TGF-βR1, which could be reversed upon silencing of KLF6 (Fig. 5E, F).

Overexpression of KLF6 in HK-2 cells stimulated with TGFβ-1 for 72 h was followed by the addition of the NF-κB inhibitor BAY-11-7082. We also indicated that KLF6 overexpression promoted IκBα phosphorylation level, as well as the expression of α-SMA, Col4α1, and TGF-βR1. Moreover, the effect of KLF6 overexpression could be reversed by BAY-11-7082 treatment (Fig. 5G). The above results indicate that KLF6 may promote the occurrence of renal cell fibrosis by activating the NF-κB signaling pathway.

### BMSC-EVs transferred miR-181d to attenuate UUO-induced renal fibrosis in rats by inactivating the KLF6-dependent NF-κB signaling pathway

We verified the effect of EV-transferred miR-181d on UUO-induced renal fibrosis in rats as a result of the activation of the KLF6/NF-κB signaling pathway. Treatment with BMSC-EVs markedly increased the expression of miR-181d, while the expression of KLF6 as well as the IκBα phosphorylation level was diminished in renal tissues of UUO rats. By contrast, treatment with BAY-11-7082 did not change the expression of miR-181d or KLF6, but there was a notable decline in the level of IκBα phosphorylation (Supplementary Fig. 2A–C). Masson’s trichrome staining and Sirius Red staining indicated a notable reduction in collagen deposition in renal tissues after treatment with either BMSC-EVs or BAY-11-7082 (Supplementary Fig. 2D, E). Western blot analysis also revealed a marked decrease in the expression of α-SMA, Col4α1, TGF-βR1, and collagen I upon treatment with either BMSC-EVs or BAY-11-7082 (Supplementary Fig. 2F). In conclusion, miR-181d transferred by BMSC-EVs can attenuate UUO-induced renal fibrosis in rats by inactivating the KLF6-dependent NF-κB signaling pathway.

## Discussion

EVs derived from MSCs appear to promote renal repair and mitigate renal disease [15]. In the present study, we found that EVs released by BMSCs contained miR-181d, which protected against the development of renal fibrosis.

We first demonstrated the successful isolation of BMSC-EVs and that they readily fused with renal tubular epithelial cells, releasing their content into the target cells. BMSC-EVs inhibited renal fibrosis both in vivo and in vitro. Previous reports showed that intraparenchymal injection of BMSCs attenuated renal fibrosis in cyclosporine-immunosuppressed rats following ischemia-reperfusion [16]. BMSCs can also suppress renal fibrosis in rats with diabetic nephropathy by inhibiting the TGF-β1/Smad3 pathway [17]. Moreover, the delivery of EVs alleviates both renal inflammation and fibrosis [18]. Consistent with our results, MSCs ameliorated renal fibrosis *via* the paracrine regulation of renal trophic factors including EVs, reflected by diminished TGF-β1 expression as well as reduced epithelial-to-mesenchymal transition of tubular epithelial cells [19]. Moreover, BMSCs-derived microvesicles protected the mouse kidney from renal injury, in part, by reducing fibrosis, interstitial lymphocyte infiltrates and tubular atrophy [20]

We also revealed that miR-181d expression was elevated in BMSC-EVs from normal renal tissues but downregulated in fibrotic renal tissues. Additionally, miR-181d expression was required for EVs derived from BMSCs to protect renal tissues from fibrosis. Ectopically expressed miR-181d inhibited renal fibrosis in vivo and in vitro, resulting in a decline in the renal fibrosis-related factors α-SMA, Col4α1, TGF-βR1, and collagen I. miR-181d was consistently reduced in both the serum of patients with renal fibrosis and in the kidneys of UUO rats. miR-181d appears to downregulate the expression of profibrotic markers including α-SMA, thereby inhibiting renal fibrosis [21]. Similarly, the elevation of miR-181d-5p was found in the serum EVs of mice treated with acupuncture with low frequency electrical stimulation, which can protect against muscle atrophy in chronic kidney disease [9]. It has been reported that EVs derived from MSCs can regulate miRNAs in renal tubular cells, favoring renal recovery [22]. A more specific study found that MSCs shuttle exogenous miR let7c *via* EVs to attenuate renal fibrosis [23], which was in line with our result. Additionally, EVs that are secreted by miR-181-5p-loaded adipose-derived MSCs were also reported to exert protection against liver fibrosis [24].

Furthermore, we found that KLF6-activated NF-κB pathway promoted renal fibrosis. miR-181d delivered by BMSC-EVs could target KLF6 in fibrosis-induced HK-2 cells, thereby inhibiting the NF-κB signaling pathway. Our luciferase activity assay validated KLF6 as a downstream target gene of miR-181d, which was further verified by RT-qPCR. A recent study by Zhang et al. has revealed that miR-181d-5p overexpression improves renal function following renal ischemia/reperfusion injury by targeted inhibition of KLF6 [25], which is partially in line with our results. Still, the methods and mechanisms in the current study are quite different from theirs. First of all, in their study, relevant detections were performed mainly at the cell level and animals. However, in our study, we determined the expression difference of miR-181d and KLF6 in renal biopsy samples from renal fibrosis patients and healthy controls through clinical case analysis, and the results were more reliable. In addition, their study conducted analysis from the perspective associated with inflammatory response, apoptosis, and renal tubular epithelial cell damage. Our study performed the bioinformatics analysis, EV isolation from BMSCs and identification, co-culture of BMSC-EVs and TGF-β1-induced HK-2 cells, investigation of the downstream factor of KLF6, detection of renal fibrosis-related proteins and construction of animal models, progressively verifying the relationship between miR-181d shuttled by BMSC-EVs and KLF6, and ultimately identifying an evolutionarily miR-181d/KLF6/NF-κB signaling in the inhibition of renal fibrosis in the context of chronic kidney disease.

Additionally, Western blot analysis and RT-qPCR demonstrated that an inhibitor of the NF-κB signaling pathway, BAY-11-7082, could counteract KLF6 function in renal fibrosis. The KLF family confers a pivotal role in physiological processes in the kidney, including the development of renal fibrosis [26]. KLF6 contributes to differentiation from nonalcoholic steatohepatitis to fibrosis from rat uncomplicated steatosis [27]. Moreover, blockade of the NF-κB signaling pathway protects renal tubular epithelial cells against renal fibrosis [28]. Consistent with our findings, KLF6 overexpression upregulated TNFα- and interleukin-1β-mediated activation of the NF-κB pathway [12]. A previous gain- and loss-of-function experiment discovered that KLF6 is responsible for lipopolysaccharide-induced pro-inflammatory gene expression and acts together with NF-κB in macrophage speciation [29]. Besides, it should be noted that EVs also contain several other substances that may participate in the progression of renal fibrosis, which is one of the limitations of the present study. For instance, a previous study has illuminated that BMSC-EVs containing MFG-E8 could repress renal fibrosis through RhoA/ROCK signaling pathway inhibition in a rat model [30]. In addition, EVs-mediated delivery of miR-21 among renal tubular epithelial cells could also trigger the progressive renal fibrosis [31].

**Fig. 6:**
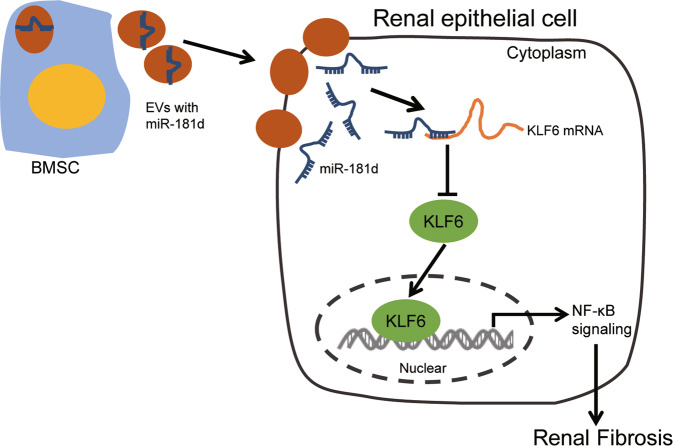
Effects of bone marrow mesenchymal stem cell-derived extracellular vesicles on renal fibrosis by regulating miR-181d/KLF6/NF-κB axis. Note: A diagram showing the mechanism whereby miR-181d is transferred by BMSC-EVs into neighboring cells where it targets KLF6, thereby inactivating the NF-κB signaling pathway and alleviating renal fibrosis in rats.

In conclusion, the major results obtained from this study revealed that miR-181d delivered by BMSC-EVs alleviates the development of renal fibrosis. miR-181d targets KLF6, which inactivates the NF-κB signaling pathway (Fig. 6). These findings suggest an inhibitory role for miR-181d-containing EVs derived from BMSCs in renal fibrosis. Nevertheless, further studies are required to explore the specific mechanism regarding KLF6-mediated NF-κB signaling pathway as well as the effect of the NF-κB signaling pathway in renal fibrosis. Also, additional studies are required for the anti-fibrogenic activities of miR-181d since published evidence has highlighted the pro-fibrogenic activities of miR-181 in chronic hepatitis B patients [32].

## Materials and methods

### Patient sample collection

Percutaneous renal biopsy samples were obtained from eight patients (Supplementary Table 2) after renal fibrosis diagnosis was confirmed. Renal biopsies from 12 patients without fibrotic lesions were used as controls (Supplementary Table 2). Patients were excluded if any of the following was present: polycystic kidney disease, pregnancy, human immunodeficiency virus, renal cancer, and recent immunosuppressive therapy.

### Animal model establishment

A total of 120 male Sprague-Dawley rats weighing 177–252 g were purchased from SLAC Laboratory Animal Co., Ltd. (Shanghai, China), and raised in a specific-pathogen-free environment. The rats were randomly divided into ten treatment groups, 12 rats per treatment (used in three animal experiments): Sham/PBS (rats receiving sham operation and injected with PBS via tail vein), Sham/EVs (rats receiving sham operation and injected with BMSC-EVs via tail vein), UUO/PBS (rats undergoing UUO surgery and injected with PBS via tail vein), UUO/EVs (rats undergoing UUO surgery and injected with 20 μg/mL BMSC-EVs via tail vein), UUO + agomir-NC (rats undergoing UUO surgery and injected with agomir-NC via tail vein) and UUO + miR-181d agomir (rats undergoing UUO surgery and injected with miR-181d agomir via tail vein), Sham (rats receiving sham operation), UUO (rats undergoing UUO surgery), UUO + BAY-11-7082 (BAY-11-7082 represents an effective and specific NF-κB inhibitor and it has been reported for repressing the inflammation of the kidney by affecting the fibrosis of kidney cells through NF-κB signaling pathway inhibition [33]). Then, eight rats from each group were randomly selected for subsequent experiments. Prior to operation, rats were anesthetized by inhalation of 2% isoflurane. UUO was performed as described previously [34]. Briefly, the left ureter was exposed by lateral incision and ligated with double bands and surgical wires. Later that day, rats were injected with BAY-11-7082 (1 mg/rat), BMSC-EVs (1 × 10^6^ per rat) or miR-181d agomir (2 mg/rat). The rats were euthanized 7 days after the first injection, and then their kidneys were fixed with 4% paraformaldehyde. The other parts were frozen in liquid nitrogen and stored at −80 °C for future use. miR-181d agomir and agomir-NC were purchased from GenePharma (Shanghai, China).

### Characterization of EVs

hBMSCs (hTERT-BMSC clone Y201), purchased from BNBIO (http://www.bnbio.com/pro/p3/16/p_35521.html), were cultured with high-glucose Dulbecco’s Modified Eagle’s medium (DMEM; Solarbio, Beijing, China) supplemented with 10% fetal bovine serum (FBS) in a 37 °C incubator with 5% CO_2_.

Flow cytometry showed that hBMSCs were positive for CD29 (ab21845), CD44 (ab25024), and CD73 (ab239246) while hematopoietic markers, such as CD34 (ab18224), CD45 (ab27287), and HLA-DR (ab1182) were absent. All antibodies were purchased from Abcam Inc. (Cambridge, MA).

### Isolation and purification of EVs

EVs were removed from FBS by centrifugation for 16 h (Beckman Coulter Avanti J-30I, Beckman Coulter Life Sciences, Brea, CA) at 100,000 × *g* and 4 °C before use in subsequent experimentation. BMSC-EVs were obtained from media after 48–72 h of incubation. EVs were isolated by ultracentrifugation at 300 × *g* for 10 min followed by 2000 × *g* for 15 min and finally 12,000 × *g* for 30 min to remove floating cells and cell fragments. Afterwards, the supernatant containing EVs was filtered through a 0.22 μm filter and then ultracentrifuged at 100,000 × *g* at 4 °C for 2 h. The pellet was washed with PBS, and the EVs resuspended. Purified EVs were immediately determined for their phenotypes and used in subsequent experiments [35]. GW4869 (an inhibitor of EV secretion; purchased from Sigma-Aldrich Chemical Company, St Louis, MO) was dissolved in 5 mM stock solution with dimethyl sulfoxide (DMSO), and then diluted to a concentration of 20 µM in the culture supernatant. After PBS washes, cells were cultured in the culture medium for 24 h, and conditioned medium was then collected [36].

### Co-culture of hBMSCs with HK-2 cells

HK-2 cells (American Type Culture Collection, VA) and BMSCs were detached with trypsin, centrifuged at 1000 × *g* for 5 min, and suspended in 3 mL DMEM. Then, the cell suspension was diluted at 1: 20 and the number of cells in 10 μL was counted. BMSCs (2 × 10^4^) were placed in the apical chambers of a 6-well plate and cultured in DMEM containing 10% serum, while HK-2 cells (3 × 10^4^) were placed in the basolateral chambers and cultured in DMEM containing 15% serum. Co-culture of BMSCs and HK-2 cells lasted for 4–5 days. The medium in the apical and basolateral chambers was replaced every 1–2 days at the same time. After 72 h and at >80% density, HK-2 cells, and BMSCs were used for following experiments.

### EV uptake

In order to determine the efficiency of BMSCs-EV uptake by HK-2 cells, EVs were labeled with green fluorescent dye (PKH67; Sigma-Aldrich), and then incubated with HK-2 cells at 37 °C for 3 h [37]. Afterwards, the cells were washed with PBS and fixed with 4% paraformaldehyde for 15 min. Next, the cell nuclei were stained with DAPI (0.5 μg/mL; Invitrogen Inc., Carlsbad, CA), and the green fluorescence in cells was observed under a fluorescence microscope.

### Cell treatment

Human renal proximal tubule epithelial cell line HK-2 (CRL-2190, American Type Culture Collection, VA) was cultured in M199 medium (Invitrogen) supplemented with 10% FBS (Gibco, Carlsbad, CA) and 2% penicillin/streptomycin in a 37 °C incubator with 5% CO_2_. The cells were then seeded in a 6-cm culture plate at a density of 1.5 × 10^6^ cells for 3–4 days. The cells were used as control or treated with 100 μg EVs for 72 h, 6 ng/mL TGF-β1 for 72 h, or 100 μg EVs + 6 ng/mL TGF-β1 for 72 h.

BMSCs were transiently transfeced according to the instructions of Lipofectamine 2000 transfection reagent (Invitrogen) with inhibitor-NC and miR-181d inhibitor, with a final concentration of 100 nM. EVs were isolated after 48 h of transfection. HK-2 cells were transiently transfected with inhibitor-NC, miR-181d inhibitor, mimic-NC or miR-181d mimic, with a final concentration of 100 nM. After 48 h of transfection, the cells were used for RT-qPCR and Western blot analysis or other experiments. Inhibitor NC, miR-181d inhibitor, mimic NC and miR-181d mimic were all purchased from GenePharma.

The lentivirus packaging system was constructed using LV5-GFP (lentivirus gene overexpression vector) and pSIH1-H1-copGFP (lentivirus shRNA fluorescent gene silencing vector). sh-KLF6-1, sh-KLF6-2, and sh-NC were synthesized by GenePharma. These vectors were co-transduced into HK-2 cells by packaging virus and target vectors, and the supernatant was collected after 48 h of culture. The supernatant contained virus particles after filtration and centrifugation, and the titer of virus was determined. According to the different transfectants, the viruses growing in the logarithmic phase were divided into oe-NC, oe-KLF6, sh-NC, sh-KLF6-1 and sh-KLF6-2. Trypsin was applied to treat and dissociate the cells when they reached logarithmic phase, and then cells were resuspended at a concentration of 5 × 10^4^ cells/mL. Next, the suspension was seeded at 2 mL per well of a 6-well plate followed by incubation overnight at 37 °C. Forty-eight hours after transduction, RT-qPCR was performed to quantify gene expression.

### RT-qPCR

TRIzol reagent (15596-018; Solarbio) was applied to extract total RNA from cultured cells or tissues in strict accordance with the instructions provided on the kit, followed by the determination of RNA concentration. The PCR primers used in this study were synthesized by Takara Biotechnology Ltd., (Dalian, China), shown in Supplementary Table 3. Reverse transcription was conducted according to the instructions of the one-step miRNA RT kit (B532451, Sangon, Shanghai, China) and complementary DNA (cDNA) RT Kit (K1622, Beijing Yaanda Biotechnology Co., Ltd., Beijing, China). Next, RT-qPCR analysis was performed on a fluorescent qPCR device (ViiA 7, Daan Gene, Guangzhou, China). mRNA expression was compared using the 2^−ΔΔCt^ relative quantification method. U6 and GAPDH served as internal references.

### Western blot analysis

High-efficiency radio-immunoprecipitation assay buffer (R0010, Solarbio) was applied to extract total protein from tissue or cells. The protein concentration of each sample was determined using a bicinchoninic acid Kit (20201ES76, Yeasen Company, Shanghai, China). Proteins were resolved by sodium dodecyl sulfate-polyacrylamide gel electrophoresis, transferred onto polyvinylidene fluoride membranes and blocked with 5% bovine serum albumin. Membranes were immunoblotted with primary rabbit polyclonal antibodies to collagen type IV alpha 1 (Col4α1; ab227616, 1:1000, Abcam), alpha-smooth muscle actin (α-SMA; ab32575, 1:1000, Abcam), transforming growth factor β receptor 1 (TGF-βR1; ab31013, 1:1000, Abcam), KLF6 (14716-1-AP, 1:1000, Proteintech, Chicago, IL), phosphorylated inhibitor of NF-κB (p-IκBα) (ab133462, 1:1000, Abcam), collagen I (ab34710, 1:1000, Abcam) as well as IκBα (CST4812, Cell Signaling Technologies, Beverly, MA) and incubated overnight at 4 °C with constant shaking. Next, the membranes were probed with horseradish peroxidase conjugated anti-rabbit against immunoglobulin G (IgG; ab205718, 1:10,000, Abcam) for 1 h. Enhanced chemiluminescence reagent was used to visualize the results by the VILBER FUSION FX5 (Vilber Lourmat, Marne-la-Vallee, France). Band intensity was quantified using the ImageJ 1.48u software (National Institutes of Health, Bethesda, MD). The relative expression of the target protein was normalized to the band intensity of GAPDH.

### Immunohistochemistry

Renal tissue blocks were fixed in 4% paraformaldehyde, followed by conventional xylene dewaxing and gradient alcohol hydration. Antigen retrieval was carried out in 0.01 M citrate buffer solution. After the addition of goat serum sealing solution, 50 μL of diluted primary antibodies against Col4α1 (ab6586, 1:200), α-SMA (ab32575, 1:100) and collagen I (ab34710, 1:1000) were added to the blocks. Next, the tissue blocks were incubated with 50 μL of goat anti-rabbit to IgG (ab6728, 1:1000) at room temperature for 1 h. Streptavidin peroxidase was added to the tissues, which were allowed to rest for 30 min at 37 °C. After diaminobenzidine coloration, the tissues were counterstained with hematoxylin and finally observed under a microscope.

### Extraction of EVs

Fluoramine (FAM)-labeled miR-181 was transfected into BMSCs and EVs were collected. The purified BMSC-EVs were mixed with 1 μm Dil lipophilic membrane stain (Invitrogen), and the EV-dye suspension was incubated for 5 min under normal mixing conditions. Excess dye was removed from the suspension by centrifugation with a 70 Ti rotor (Beckman Coulter Life Sciences, Brea, CA) for 1 h at 100,000 × *g* and 4 °C. Next, the precipitate containing EVs was resuspended in PBS and washed three times. After the last spin, precipitates were resuspended in PBS. The EVs labeled with Dil were cultured with HK-2 cells for 6 h, after which the HK-2 cells were fixed in 4% paraformaldehyde. The uptake was observed under a fluorescence microscopy.

### Masson’s trichrome staining

Renal tissue sections were stained with hematoxylin solution and then stained in Biebrich scarlet acid fuchsin solution. Following this, the sections were differentiated in phosphotungstic acid solution. Once the collagen-rich area lost red color and became clear, observed by naked eyes, the next step was conducted. The slide was transferred directly into the aniline blue solution and stained for 5–10 min, followed by differentiation in 1% acetic acid solution for 2–5 min. Thereafter, the sections were dehydrated with 95% alcohol and anhydrous alcohol, cleared in xylene, mounted with resin mounting agent and observed under an optical microscope.

### Sirius red staining

Sirius Red staining was performed to detect collagen. The sections were deparaffinized, hydrated and washed. Next, the sections were soaked in 1% Sirius Red/saturated picric acid solution for 1 h, then rinsed with 0.5% acetic acid, dehydrated and sealed with mounting agent before observation under an optical microscope.

### In situ hybridization

In situ hybridization was performed following the method described in a previous study [38]. The 3% H_2_O_2_ was used to quench the endogenous peroxidase activity. After treatment with protease K, the slides were fixed in 4% paraformaldehyde. The slides were incubated in hybrid buffer at 60 °C for 2 h, and then incubated overnight with wild-type miR-181d or control probes containing a mutated miR-181d sequence (50 nm; digitalis glycoside labeled locked nucleic acids probe, Exiqon, Vedbaek, Denmark). Alkaline phosphatase substrate was used for alkaline phosphate reaction, and the slide was mounted using a water-based solid sealing agent. Images were taken with an Olympus bx-60.

### Dual luciferase reporter gene assay

The biological prediction website, Starbase, was utilized to predict the binding sites between KLF6 and miR-181d, which were confirmed using dual luciferase reporter gene assays. The potential miR-181d binding site sequence in the KLF6 3′untranslated region (3′UTR) and a corresponding mutated sequence were cloned into the pGLO vector creating the PGLO-KLF6-WT and PGLO-KLF6-MUT reporter plasmids. The two reporter plasmids were co-transfected into HEK-293T cells with miR-181d mimic- or mimic-NC plasmids (Invitrogen). The supernatant of HEK-293T cells was collected 24 h later. Luciferase activity was measured using dual luciferase reporter gene detection system (E1910, Promega Corp., Madison, WI). The relative luciferase activity was normalized by determining the ratio of the firefly luciferase activity to that of Renilla luciferase [39].

### Statistical analysis

Data analysis was performed utilizing SPSS 21.0 statistical software (IBM Corp., Armonk, NY). Measurement data were expressed as mean ± standard deviation. The data obeying normal distribution and homogeneous variance in independent design between two groups were compared using independent sample *t* test. Data among multiple groups were compared using one-way analysis of variance, followed by a post hoc correction using Tukey’s test. A value of *p* < 0.05 was indicative of a statistically significant difference.

## Supplementary information

original western blots

Supplemental Material

aj-checklist

## Data Availability

The datasets generated and/or analysed during the current study are available in the paper and Supplementary Materials.
