# Supplementary material for: Bone marrow mesenchymal stem cell-derived extracellular vesicles containing miR-181d protect rats against renal fibrosis by inhibiting KLF6 and the NF-κB signaling pathway
Source: Cell Death Dis. 2022 Jun 7;13(6):535. doi: 10.1038/s41419-022-04875-w (PMC9174332; doi:10.1038/s41419-022-04875-w)
Supplement: Supplementary file 1 — original western blots [file 41419_2022_4875_MOESM1_ESM.docx]

Figure 1F

















Figure 3G
















Figure 4G







Figure 5F
