# Supplementary material for: Bone marrow mesenchymal stem cell-derived extracellular vesicles containing miR-181d protect rats against renal fibrosis by inhibiting KLF6 and the NF-κB signaling pathway
Source: Cell Death Dis. 2022 Jun 7;13(6):535. doi: 10.1038/s41419-022-04875-w (PMC9174332; doi:10.1038/s41419-022-04875-w)
Supplement: Supplementary file 2 — Supplemental Material [file 41419_2022_4875_MOESM2_ESM.docx]

**Supplementary table 1** Correlation and core degree of the top 10 genes related to KLF6 expression.

| Gene | Core | Core *p* value | Degree | Sum combined score |
| --- | --- | --- | --- | --- |
| KLF6 | 1 | 0 | 5 | 2.035 |
| RHOU | 0.979 | 2.27E-15 | 4 | 1.06 |
| IL15 | -0.979 | 2.37E-15 | 6 | 1.709 |
| JUNB | 0.975 | 1.32E-14 | 6 | 3.381 |
| EGR1 | 0.971 | 6.24E-14 | 7 | 3.839 |
| EGR2 | 0.971 | 7.47E-14 | 6 | 3.065 |
| NFKB1 | 0.971 | 7.92E-14 | 7 | 2.612 |
| JUN | 0.97 | 1.06E-13 | 8 | 5.463 |
| SLC25A11 | -0.968 | 1.84E-13 | 0 | 0 |
| CLCF1 | 0.968 | 1.85E-13 | 1 | 0.201 |
| ARID5A | 0.967 | 2.47E-13 | 2 | 1.201 |

**Note:** “Core” represents the strong or weak correlation between gene and KLF6 expression; “core *p* value” represents the *p* value indicating the statistically significant correlation between the gene and KLF6 expression; “degree” represents the core degree of genes in the PPI network, that is, the number of interaction relationship between the gene and other genes; “sum combined score” represents the total score of interaction between gene and other genes in the PPI network.

**Supplementary table 2** Demographics of patients with renal fibrosis and controls without fibrotic lesions

| Variables | Normal | Renal fibrosis | *p* |
| --- | --- | --- | --- |
| Sex |  |  | 0.852 |
| Male | 7 | 5 |  |
| Female | 5 | 3 |  |
| Age | 46.67 ± 13.27 | 48.38 ± 15.06 | 0.792 |
| β2-MG (mg/L) | 1.34 ± 0.37 | 6.16 ± 0.67 | < 0.0001 |
| GFR (ml/min/L) | 103.51 ± 8.27 | 83.62 ± 27.65 | 0.0052 |
| CKD stage (I/II/III/IV) | - | 2/3/2/1 | - |

**Supplementary table 3** Primer sequences for RT-qPCR

| Gene | Primer sequence (5'-3') |
| --- | --- |
| miR-181d [human & rat] | F: 5’-CGGCGGAACATTCATTGTTGTCG-3’ |
|  | R: Universal reverse primer |
| KLF6 [human] | F: 5’-AACCAGGCACTTCCGAAAGCAC-3’ |
|  | R: 5’-CTCAGAGGTGCCTCTTCATGTG-3’ |
| COL4α1 [human] | F: 5’-TGTTGACGGCTTACCTGGAGAC-3’ |
|  | R: 5’-GGTAGACCAACTCCAGGCTCTC-3’ |
| α-SMA [human] | F: 5’-CTATGCCTCTGGACGCACAACT-3’ |
|  | R: 5’-CAGATCCAGACGCATGATGGCA-3’ |
| TGF-βR1 [human] | F: 5’-GACAACGTCAGGTTCTGGCTCA-3’ |
|  | R: 5’-CCGCCACTTTCCTCTCCAAACT-3’ |
| U6 | F: 5’-CTCGCTTCGGCAGCACA-3’ |
|  | R: Universal reverse primer |
| GAPDH | F: 5’-CCCACTCCTCCACCTTTGAC-3’ |
|  | R: 5’-CATACCAGGAAATGAGCTTGACAA-3’ |

**Note:** RT-qPCR, reverse-transcription quantitative polymerase chain reaction; miR, microRNA; KLF6, krüppel-like factor 6; COL4α1, collagen type IV alpha 1; α-SMA, alpha-smooth muscle actin; TGF-βR1, transforming growth factor β receptor 1; F, forward; R, reverse

**
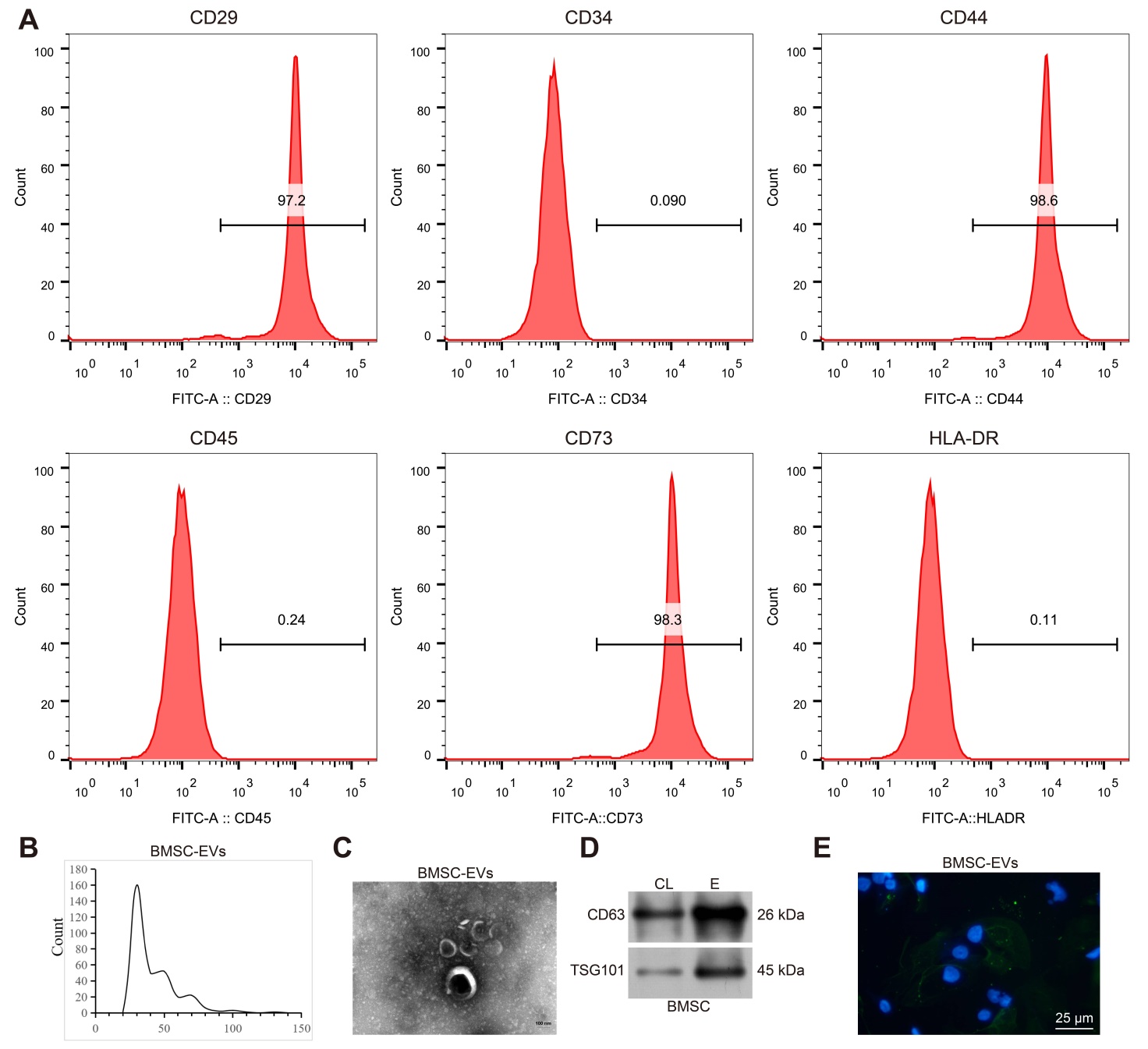
**

**Supplementary Figure 1** Characterization of the isolated BMSC-EVs. a, BMSCs surface markers (CD29, CD44, CD73, CD34, CD45, and HLA-DR) were assessed by flow cytometry. b, EV size distribution as measured by dynamic light scattering. c, Transmission electron microscopic observation of EVs. d, Western blot analysis of EV maker proteins CD63 and TSG101 (CL: cell lysate; E, EVs). e. Fluorescence microscopic observation of PKH-labeled EVs internalized by HK-2 cells (green fluorescence: PKH67-labeled EVs, blue fluorescence: DAPI-labeled HK-2 cells).

**
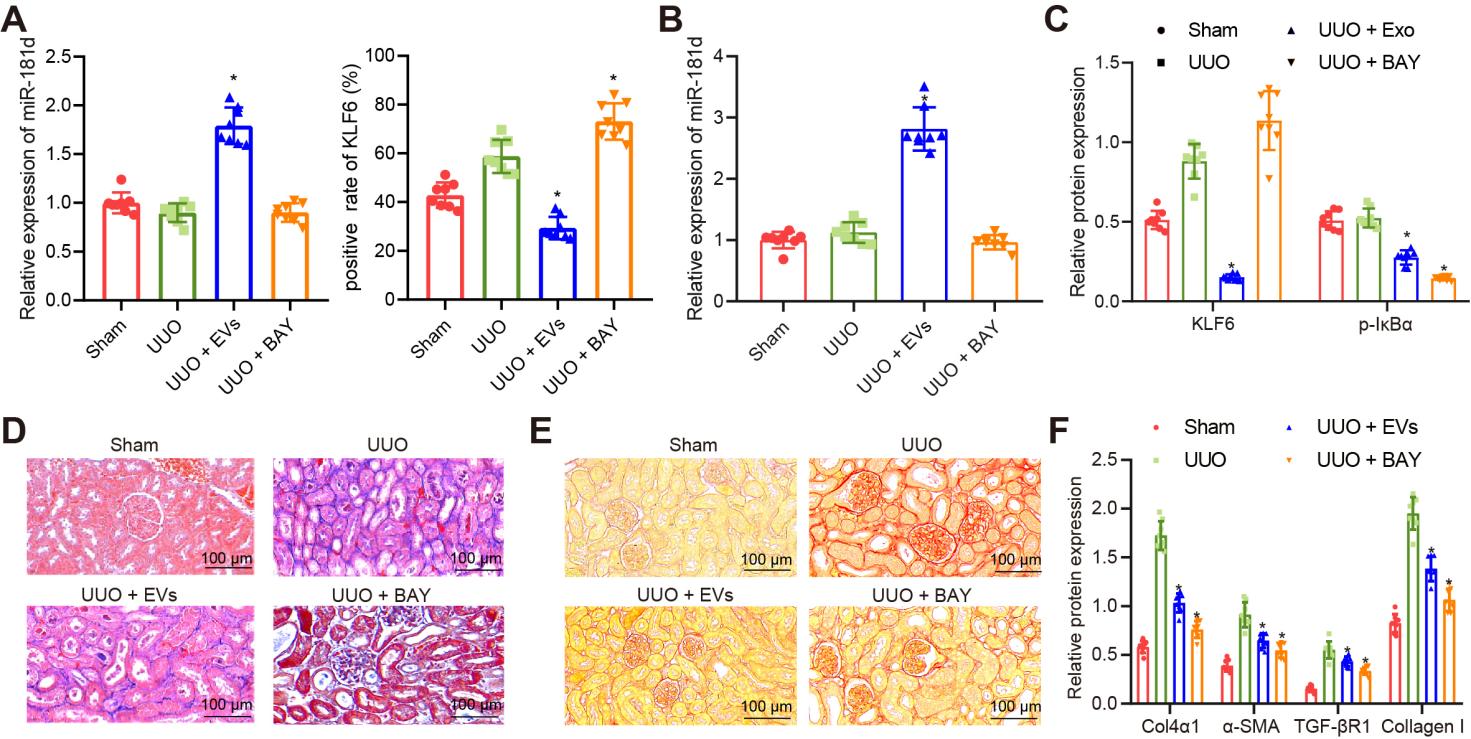
**

**Supplementary Figure 2** miR-181d delivered by BMSC-EVs attenuates UUO-induced renal fibrosis in rats by disrupting the KLF6-dependent NF-κB signaling pathway activation. a, *In situ* hybridization and immunohistochemical assay for miR-181d and KLF6 expression in renal tissues of UUO rats treated with BMSC-EVs or BAY-11-7082. b, RT-qPCR analysis of miR-181d mRNA expression in renal tissues from UUO rats after being injected with BMSC-EVs or BAY-11-7082. c, Western blot analysis of KLF6 protein expression as well as the level of IκBα phosphorylation in renal tissues of UUO rats after being injected with BMSC-EVs or BAY-11-7082. d, Masson's trichrome staining for collagen deposition in renal tissues from UUO rats treated with BMSC-EVs or BAY-11-7082. e, Sirius Red staining of collagen deposition in renal tissues from UUO rats treated with BMSC-EVs or BAY-11-7082. f, Western blot analysis of Col4α1, α-SMA, TGF-βR1 and collagen I protein expression in renal tissues from UUO rats treated with BMSC-EVs or BAY-11-7082. * *p* < 0.05 *vs*. UUO rats. n = 8.
